# Lymphatico-venous anastomosis as treatment for breast cancer-related lymphedema: a prospective study on quality of life

**DOI:** 10.1007/s10549-017-4180-1

**Published:** 2017-03-07

**Authors:** Anouk J. M. Cornelissen, Melissa Kool, Tiara R. Lopez Penha, Xavier H. A. Keuter, Andrzej A. Piatkowski, E. Heuts, René R. W. J. van der Hulst, Shan Shan Qiu

**Affiliations:** 1 Department of Plastic and Reconstructive Surgery, Maastricht University Medical Center, P. Debyelaan 25, 6229 HX Maastricht, The Netherlands; 2 Department of Surgery, Maastricht University Medical Center, P. Debyelaan 25, 6229 HX Maastricht, The Netherlands

**Keywords:** Breast cancer, Lymphedema, Quality of life, Lymphatico-venous anastomosis

## Abstract

**Purpose:**

Lymphedema is a chronic and disabling sequel of breast cancer treatment that can be treated by lymphatico-venous anastomosis (LVA). Artificial connections between the venous and lymphatic system are performed supermicrosurgically. This prospective study analyses the effect of LVA on quality of life.

**Methods:**

A prospective study was performed between November 2015 and July 2016 on consecutive patients in the Maastricht University Medical Centre. Quality of life was considered as the primary outcome, and the Lymphedema International Classification of Functioning (Lymph-ICF) questionnaire was used. Discontinuation of compressive stockings and arm volume, using the Upper Extremity Lymphedema index (UEL-index), were the secondary outcomes.

**Results:**

Twenty women with early-stage breast cancer-related lymphedema (BCRL) were included. The mean age was 55.9 ± 4 years and the median BMI was 25.1 [21–30] kg/m^2^. The mean follow-up was 7.8 ± 1.5 months. Statistically significant improvement in quality of life was achieved in the total score and for all the quality of life domains after one year of follow-up (*p* < 0.05). The discontinuation rate in compressive stockings use was 85%. The difference in mean relative volume did not show a statistically significant decrease.

**Conclusions:**

LVA for early-stage BCRL resulted in a significant improvement in quality of life and a high rate in stocking discontinuation.

## Purpose

Lymphedema is a debilitating disease with a profound adverse effect on quality of life [1]. Breast cancer-related lymphedema (BCRL) is caused by an acquired interruption of lymphatic drainage after loco regional treatment such as radiotherapy and/or lymph node dissection [2–5]. Since breast cancer survival rates have risen over the past decades, there is an increasing demand by patients to focus on the improvement of quality of life after breast cancer [6, 7]. Therefore, the search for effective treatments in BCRL has gained popularity.

Standard therapy for lymphedema remains ‘complex decongestive therapy’, which aims to ameliorate the symptoms and delay progression of this chronic disease [8, 9]. However, for most patients a lifelong, complex decongestive therapy might be necessary.

Supermicrosurgical techniques, such as lymphatico-venous anastomosis (LVA), have been used with satisfactory results since the introduction of this technique by Koshima in the late ‘90 s [10–13]. By means of an LVA, an artificial connection is created between a patent lymphatic collector and a subdermal vein in a lymphoedematous limb. This principle is based on the existence of physiological anatomic connections between the lymphatic and the venous system [14–17].

Recent advances in techniques, such as the indocyanine green (ICG) fluorescence lymphangiography, more precise microscopes and optimal patient selection have resulted in less invasive and faster LVA procedures, allowing LVA to be performed under local anaesthesia with a substantially reduced operating time [18–22].

In previous studies, reduction in volume was mostly named as the primary outcome after lymphedema treatment [10–13]. Yet an improvement in quality of life should be considered as the ultimate aim in treating BCRL patients. Therefore, this current prospective study evaluates the effect of LVA on the quality of life of patients with BCRL.

## Methods

### Patient selection

A prospective study was performed, including consecutive patients between November 2015 and July 2016 in the Maastricht University Medical Centre. Inclusion criteria consisted of an evidenced upper limb lymphedema secondary to breast cancer in stage 1 or 2A according to the International Society of Lymphology (ISL) classification [23], patent lymphatic ducts seen by ICG lymphangiography and an absence of skin infections and complex decongestive therapy for at least 3 months.

Demographic data and other factors that might influence lymphedema such as chemotherapy, radiotherapy, lymph node dissection and previous skin infections, were documented. Complications secondary to the surgery were registered.

### ICG lymphangiography

Preoperatively, all patients underwent ICG lymphangiography to determine whether patent lymphatic ducts could be tracked. ICG 0.5%, 0.05 ml (PULSION^®^ 25 mg for solution, PULSION Medical Systems SE, Feldkirchen, Germany) was subcutaneously injected in the second and fourth web spaces. After 15–30 min, the fluorescence was detected using a near infrared camera (Fluobeam^®^; Fluoptics, Grenoble France).

### Surgical technique

The operation was performed (SQ and RH) using the technique described by Koshima et al. [24] under local anaesthesia (bupivacaine hydrochloride 5 mg/ml with adrenaline 5μg/ml). In brief, the lymphatic ducts were located and an anastomosis was made with a subcutaneous vein using Ethilon 11-0. Patency was checked intraoperatively by distal injection of methylene blue in all cases, and when possible, by ICG lymphangiography after finishing the anastomosis. Patients were told not to use stockings or conservative treatment in the first month after surgery to prevent damage of the fragile, newly performed anastomosis.

### Outcomes

Quality of life was considered as the primary outcome, measured by the Lymphedema international classification of functioning (Lymph-ICF) questionnaire (Dutch version) [25]. This questionnaire comprises five domains: physical function, mental function, household activities, mobility activities and life and social activities. The values range from 1 to 100; a lower score on the questionnaire indicates a better quality of life. Patients were asked to fill in the questionnaires at 1, 3, 6 and 12 months postoperatively with a time window of 4 weeks. A decrease in the VAS score of more than 11, and an increase of more than 9 are considered to be statistically significant (*p* < 0.05) as validated previously [25].

Secondary outcomes were the use of compressive stockings and arm volume changes according to the Upper Extremity Lymphedema index (UEL-index) [26]. Measurements were obtained preoperatively and at 1, 3, 6 and 12 months postoperatively by the same independent researcher (AC) to prevent inter-observer bias.

### Statistical analysis

Continuous variables were reported as mean with standard deviation or median with range, depending on the normality tested by the Kolmogorov–Smirnov test. Categorical data were reported as frequencies and proportions. Statistically significant differences in mean between groups were tested using a paired two-tailed *t* test. A *p* value < 0.05 was considered to be statistically significant. Results were analysed using SPSS Statistics 24.

## Results

### Study characteristics

A total of 20 patients with BCRL were included. Stage 1 according to ISL was present in one patient, whereas stage 2A was seen in the rest of the patients. All these patients were female with a mean age of 55.9 ± 4 years and a median BMI of 25.1 [21–33] kg/m^2^. The median time since the onset of arm lymphedema was 6 [2–30] years and 25% of the patients had experienced at least one episode of skin infection since the diagnosis of lymphedema. The mean follow-up time was 7.8 ± 1.5 months. Information on main study charactersitcs are shown in Table 1.

**Table 1:** The main study characteristics are presented. Patient characteristics, type of cancer treatment and characteristics of the surgery

Characteristics	Outcome
Patient characteristics	
Number of patients	20
Female	20
Age	55.9 ± 4 years
BMI	25.1 [21–30] kg/m^2^
Time from lymphedema onset	6 [2–30] years
Mean follow-up	7.8 ± 1.5 months
Type of treatment	
Chemotherapy	85%
Radiotherapy	75%
Lymph node dissection	100%
Mean number of Lymph nodes dissected	15.9
Mean number of positive lymph nodes	4.2
Surgery characteristics	
Mean operating time	92 ± 8 min
Mean number of anastomosis	1.5

The average operating time was 92 ± 8 min. The number of anastomoses per patient varied between one and two, and no statistically significant difference was found. Minor complications were observed postoperatively in two cases due to skin irritation at the site of the contrast injection. These complications were resolved with a conservative approach.

### Outcomes

**Fig. 1:**
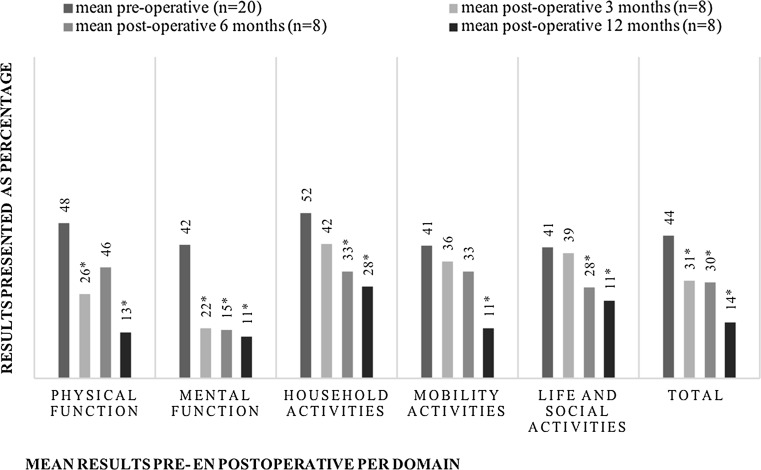
The mean outcome on the VAS score pre- and postoperative according to the Lymph-ICF preoperatively and after 3, 6 and 12 months. * indicates a statistical significant decrease compared to the preoperative measurement. *n* number of patients who filled in the questionnaire at that time of follow-up

Regarding quality of life, all the domains and the total score of the Lymph-ICF showed improvement postoperatively. In comparison with the preoperative results, statistical significance was encountered for all the domains and the total score after one year of follow-up (*p* < 0.05). (Figure 1)

The discontinuation of compressive stocking use was achieved in 85% (*n* = 17) of patients.

The mean relative volume difference in UEL between a healthy and lymphoedematous arm preoperatively was 14.92 ± 8.01 and postoperatively 12.99 ± 7.47. However, the difference did not reach statistical significance (*p* = 0.582).

## Discussion

Lymphedema might be considered as one of the main complications of breast cancer treatment, with a crucial impact on the quality of life of breast cancer survivors. Therefore, the main goal in treating lymphedema patients should be an improvement in their quality of life. In the current prospective study, according to the pre- and postoperative QOL questionnaires, a substantial improvement was achieved after performing LVA procedures within the first-year follow-up.

It was remarkable that the total score, obtained via the questionnaires, showed a statistically significant difference, suggesting that through this surgical procedure, quality of life can be improved. The current study reported a statistically significant QOL improvement in 90% of the cases. In those patients with no significant difference, the preoperative scores were low, which might indicate that the lymphedema, even though clinically evident, did not have a relevant impact on their quality of life. However, in none of the cases did the total score of quality of life deteriorate after surgery. The physical function presented a statistically significant improvement after surgery. This domain evaluates the ‘classic’ lymphedema symptoms like pain, stiffness, swelling, loss of strength, tingling and tenseness. The majority of patients stated that a less disagreeable feeling was experienced in their arm postoperatively, mainly due to less heaviness and partially because of the discontinuation of compressive stockings. The improvement in physical symptoms could have led to a betterment in mobility, household activities and life and social activities. These three domains improved statistically and significantly only after 6 and/or 12 months, but not immediately after the surgery. Significant differences were also present in the mental function of the patients after surgery. As mentioned before, the use of stockings was considered as one of the patients’ predominant complaints. Without stockings, the lymphedema may become less obvious to the patients’ surroundings, thus enhancing their self-confidence and self-control about the disease.

Previous studies achieved similar percentages in the subjective symptoms after LVA (61.5–100%) [11–13, 27–29]. A wide variety of methods were used to measure these subjective symptoms, such as retrospective interviews [30], scale scoring systems for subjective pre- and postoperative complaints [31, 32] or subjective assessment by a skin therapist [33]. Masia et al. [34] and Chen et al. [35, 36] used two different, standardised self-developed questionnaires on lymphedema-related quality of life and reported an improvement in 100% of their patients. The drawbacks were that no detailed results per category were available on these scales, and neither were they developed according to the three stages recommended for patient reported outcome measures (PROM) nor validated according to those standard stages. Therefore, no statistical analysis on significance could be performed. Moreover, the questionnaire used by Chen included only the physical function, which represents only one of the five domains of the Lymph-ICF. In contrast, the Lymph-ICF is validated and reproducible, allowing to systematically measure the changes resulting from surgery. The Lymph-ICF questionnaire gives a score on five different domains, which may provide a wider coverage of all the aspects related to lymphedema [25]. This questionnaire can determine changes over time and may provide useful and detailed information for long-term follow-up. Furthermore, the used questionnaire is the only lymphedema-related questionnaire that uses a VAS score answering model, which is more sensitive to subtle changes. In addition, the existence of a different version of the same questionnaire for lower limb lymphedema is interesting. Since the impact of quality of life may differ depending on the location of this disease, having two different versions might allow for a more accurate assessment of the results. Although a questionnaire aims to objectify subjective symptoms, the blinding of the patients might not be possible since it is an self-completed questionnaire.

The current study demonstrated a high discontinuation rate in the use of compressive stockings after surgery. This might be explained by the release of arm heaviness as indicated by the patients in the questionnaire. Patients reported an immediately noticeable effect after the surgery. This improvement was maintained during the follow-up period, and patients felt that stockings were no longer needed in order to control the volume of their arms. Even though the use of stockings represented one of the most deleterious factors for their quality of life, only few studies have reported their results on stocking discontinuity. A recent literature review performed by Basta et al. reported a discontinuation of compression therapy of 56.3% in the previous studies reporting on LVA in upper and lower limb [12].

In contrast with the good rate of quality of life improvement and stocking discontinuity, the volume reduction did not show a proportional change in the present study. A non-statistically significant reduction in excess limb volume of 12.94% was observed in comparison with previously reported rates of 42.9–64.6% [37–40]. This difference in results might be explained by the fact that 80% (*n* = 16) of the patients in this study came to the preoperative outpatient clinic visit wearing their stockings. Within the group of patients who ceased to use stockings after surgery, 50% (*n* = 10) showed an increase in arm volume. The fact that all these patients wore stockings during the preoperative measurements should be taken into account. The other 50% (*n* = 10) of the patients did show a volume reduction. Of these patients, only 15% (*n* = 5) wore their stockings during preoperative measurements.

The improvement in quality of life and the high rate of stockings cessation suggest that the LVA procedure may be successful in reducing complaints related to BCRL by preventing an accumulation of lymph fluid in the interstitial tissue [14–17]. Objective measurements alone might not be sufficient to assess the improvement in other factors such as the sensation of heaviness, and unstable volume of the arm or pain. For this purpose, the use of quality of life questionnaires should be encouraged to properly assess the impact of this disease and its treatments on patients.

## Conclusions

LVA performed under local anaesthesia in patients with early-stage BCRL resulted in a high rate of stocking discontinuation, contributing significantly to an improvement in the quality of life of breast cancer survivors.
